# The influence of habitat on the evolution of plants: a case study across Saxifragales

**DOI:** 10.1093/aob/mcw160

**Published:** 2016-08-22

**Authors:** Rafael Rubio de Casas, Mark E. Mort, Douglas E. Soltis

**Affiliations:** ^1^Estación Experimental de Zonas Áridas, EEZA-CSIC, Carretera de Sacramento s/n, 04120 Almería, Spain; ^2^CEFE UMR 5175, CNRS, Universite de Montpellier, Universite Paul-Valery 7 Montpellier, EPHE, 1919 route de Mende, 34293 Montpellier cedex 05, France; ^3^Departamento de Ecología, Facultad de Ciencias, Universidad de Granada, Avda. de la Fuentenueva s/n, 18071 Granada, Spain; ^4^Department of Ecology and Evolutionary Biology and Biodiversity Institute, University of Kansas, 1200 Sunnyside Avenue, Lawrence, KS 66045-7543, USA; ^5^Department of Biology, University of Florida, Gainesville, FL 32611, USA; ^6^Florida Museum of Natural History, University of Florida, Gainesville, FL 32611, USA

**Keywords:** *Aeonium*, anagenesis, cladogenesis, Crassulaceae, climate change, diversification, ecotone, habitat selection, Macaronesia, niche conservatism, *Saxifraga*

## Abstract

**Background and Aims** Organismal evolution tends to be closely associated with ecological conditions. However, the extent to which this association constrains adaptation or diversification into new habitats remains unclear. We studied habitat evolution in the hyper-diverse angiosperm clade Saxifragales.

** Methods** We used species-level phylogenies for approx. 950 species to analyse the evolution of habitat shifts as well as their influence on plant diversification. We combined habitat characterization based on floristic assignments and state-of-the art phylogenetic comparative methods to estimate within- and across-habitat diversification patterns.

** Key Results** Our analyses showed that Saxifragales diversified into multiple habitats from a forest-inhabiting ancestor and that this diversification is governed by relatively rare habitat shifts. Lineages are likely to stay within inferred ancestral ecological conditions. Adaptation to some habitat types (e.g. aquatic, desert) may be canalizing events that lineages do not escape. Although associations between increased diversification rates and shifts in habitat preferences are occasionally observed, extreme macroevolutionary rates are closely associated with specific habitats. Lineages occurring in shrubland, and especially tundra and rock cliffs, exhibit comparatively high diversification, whereas forest, grassland, desert and aquatic habitats are associated with low diversification.

** Conclusions** The likelihood of occupation of new habitats appears to be asymmetric. Shifts to aquatic and desert habitats may be canalizing events. Other habitats, such as tundra, might act as evolutionary sources, while forests provide the only habitat seemingly colonized easily by lineages originating elsewhere. However, habitat shifts are very rare, and any major environmental alteration is expected to have dramatic evolutionary consequences.

## INTRODUCTION

Modern evolutionary ecology has been strongly influenced by the idea that most diversification tends to occur within ancestral ecological conditions. This principle, termed phylogenetic niche conservatism (PNC), results in related species occurring in similar environments (Harvey and Pagel, 1991; Wiens and Graham, 2005; Donoghue, 2008; Crisp and Cook, 2012). However, it is undeniable that biome shifts and the colonization of new habitats have also been prevalent throughout evolutionary history (Donoghue and Edwards, 2014)

To date, we lack the capacity to define clearly the environmental or functional factors constraining ecological shifts at an evolutionary scale (Crisp and Cook, 2012). However, anticipating the rate of adaptation to and diversification into new environments seems crucial to apprehend the evolutionary consequences of global change (Parmesan, 2006). It has been argued that current human-induced climate change might lead to widespread extinction because it outpaces both the rate of adaptation *in situ* and the ability of organisms to track moving habitat boundaries (Corlett and Westcott, 2013; Kubisch *et al.*, 2013; Quintero and Wiens, 2013). At this point, however, we cannot make general predictions about the consequences of habitat changes for the conservation of biodiversity above the species level (Wiens *et al.*, 2010). In this context, analyses of past habitat shifts might provide valuable insights into the evolutionary significance of future perturbations (Donoghue, 2008; Wiens *et al.*, 2010).

Increasing temperatures and changes in rainfall seasonality are predicted to affect the relative expansion or contraction of habitat types across ecotones (Van Auken, 2000; Parmesan, 2006; Hirota *et al.*, 2010; He *et al.*, 2015). Variable environmental boundaries are also expected to be highly consequential to the distribution and range of discontinuous habitats such as wetlands (Hamilton, 2010; Oberlander *et al.*, 2014; Saintilan and Rogers, 2015). Anticipating the biodiversity consequences of these changes in habitat distribution and availability requires the capacity to estimate the likelihood of the lineages occupying each habitat to adapt to new conditions and/or shift into surrounding environments.

Organisms exhibit a general tendency to retain traits associated with ancestral ecological conditions throughout evolution (Harvey and Pagel, 1991; Holt and Gaines, 1992). This implies that habitat shifts are more likely to occur between habitats with overlapping environmental conditions than between more extreme habitats. It also implies that the transitions to and from habitats requiring a higher degree of specialization will be less frequent, whereas ‘intermediate’ habitats might facilitate the transition among more extreme habitats. In particular, environments such as deserts and aquatic habitats might require very specific suites of adaptations that can be hypothesized to limit the rates of colonization by and diversification of angiosperms (Stebbins, 1974; Cook, 1999; Guerrero *et al.*, 2013).

From a phylogenetic perspective, adaptation to a new habitat can be described as an anagenetic habitat shift (i.e. a change along a branch of the tree that results in habitat replacement). However, in many cases, habitat changes will not affect all populations of a given taxon equally (Harte *et al.*, 2004). Some populations might remain within the ancestral environment while others may face more drastic changes (Bellard *et al.*, 2012). As a result, some daughter taxa will not shift habitat while others will, thereby undergoing a cladogenetic habitat shift. Whether past habitat shifts have been associated with cladogenesis, anagenesis or extinction might be indicative of the evolutionary consequences of environmental change for a given group (Condamine *et al.*, 2013).

In the case of plants, certain habitat shifts appear to be particularly important from an evolutionary perspective (Stebbins, 1974; Donoghue and Edwards, 2014). For instance, ecotones such as forest/tundra, grassland/forest, shrubland/forest, grassland/shrubland and shrubland/desert have been found to be important from a biogeographical and phylogenetic perspective. Many plant groups appear to have originated in forest environments and later adapted to and colonized drier, more disturbed and colder habitats. This ‘out of the forest’ transition appears to have been a key step for the formation of tundra, grasslands and Mediterranean shrublands (i.e. chaparral; Murray, 1995; Verdú *et al.*, 2003; Ackerly, 2004; Edwards and Smith, 2010; Brochmann *et al.*, 2013). On the other hand, several grassland lineages seem to have originated in drier, shrubland-like environments (Lamont *et al.*, 2013). This same kind of habitat seems to have been occupied by the ancestors of several desert lineages (Heibl and Renner, 2012; Pittermann *et al.*, 2012; Guerrero *et al.*, 2013). Consequently, it can be hypothesized that when comparing the likelihood of habitat shifts, it should be easier to find evidence for evolutionary shifts from forest habitats into tundra, grasslands or shrubland and from shrubland into grasslands and deserts than the reverse.

Studying the influence of habitat associations in the evolution of a group of organisms requires that the group exhibit wide phylogenetic diversity, with several independent lineages occurring in different habitats. Moreover, a robust, dated phylogenetic hypothesis including taxa that occur across all of the habitats should be available.

Saxifragales are one such group that permit rigorous analysis of habitat associations in a phylogenetic context. Saxifragales *sensu* APG III (2009; APG IV 2016 was published after our study was complete) recognized 15 families in Saxifragales: Altingiaceae, Aphanopetalaceae, Cercidiphyllaceae, Crassulaceae, Dap-hniphyllaceae, Grossulariaceae, Haloragaceae Hamamelidaceae, Iteaceae, Paeoniaceae, Penthoraceae, Peridiscaceae, Pterostemonaceae, Saxifragaceae, and Tetracarpaeaceae. This angiosperm clade of approx. 2500 species has a rich fossil record and is hyper-diverse morphologically, including trees, shrubs, lianas, annual and perennial herbs, succulents and aquatics that occur in a wide variety of habitats, including forest, aquatic, grasslands, desert shrubland and tundra. No other clade of angiosperms of comparable size harbours so much diversity.

Both molecular data and the fossil record suggest that the diversification of Saxifragales was rapid and provide a well-defined time frame for the evolution of the group. Fossils indicate that Saxifragales was once more diverse and widespread than the extant members suggest and that the group encompassed wide phenotypic diversity early in its history (Fishbein *et al.*, 2001; Jian *et al.*, 2008). Jian *et al.* (2008) estimated the origin and subsequent diversification of Saxifragales as between 112 (± 9·7) and 120 (± 10·2) million years ago, with the major lineages within the clade appearing in as little as 3–6 million years ago. Moreover, the phylogenetic relationships of Saxifragales have been analysed in detail, and a well-resolved large tree covering much of the extant species diversity is already available (Jian *et al.*, 2008; Soltis *et al.*, 2013). The wealth of available information makes Saxifragales one of the best sampled and resolved large clades and thus an ideal case study for detailed, large-scale evolutionary ecology analyses.

We have used the rich data available for Saxifragales and recently developed tools for macroevolutionary analysis to explore the effect of habitat specialization and habitat shifts on plant diversification. Our aims were to investigate the consequences for speciation and extinction associated with specific habitats and to explore the probabilities of diversification across habitat borders. We focused on five habitat boundaries that have been reported as important for angiosperm evolution and to be likely to shift as a consequence of climate change: forest/tundra, grassland/forest, shrubland/forest, grassland/shrubland and shrubland/desert. We addressed these objectives by analysing: (*a*) the evolutionary history of habitat occupation in Saxifragales, to determine the habitat of the most recent common ancestor (MRCA) of the group; (*b*) the relative diversification rates associated with each habitat type and the consequences for diversification of habitat shifts, whether anagenetic or cladogenetic; and (*c*) the evolutionary dynamics across the five focal habitat boundaries (i.e. the relative likelihood of lineages in each habitat to colonize the other).

## MATERIALS AND METHODS

### Phylogenetic hypothesis and habitat coding

The phylogeny used in all analyses was the maximum likelihood (ML) tree described in Soltis *et al.* (2013). Briefly, this tree is a phylogeny of Saxifragales at the species level that contains 950 operational taxonomic units (OTUs) – 36·8 % of species-level diversity of the clade. The tree was obtained using a supermatrix approach and ML estimations. Branch lengths were later calculated using penalized likelihood as implemented in r8s (Sanderson, 2002, 2003) with the time constraints described in Jian *et al.* (2008).

Determining the habitat characteristics of large taxonomic sets is problematic because sampling effort is necessarily different among species (i.e. not all species are equally well sampled across their range). Moreover, quantitative habitat characterizations of biological niches rely on distributional data stored in repositories such as the Global Biodiversity Information Facility (GBIF) and environmental characterizations based on modelled climatic values such as the BIOCLIM WorldClim variables. These large-scale approaches are very powerful and have been used successfully to infer climate-related evolution in several cases (e.g. Zanne *et al.*, 2014; Wüest *et al.*, 2015). However, these approaches can be easily mired by erroneously or incompletely labelled accessions and poor global sampling (Yesson *et al.*, 2007; Feeley and Silman, 2010; Edwards *et al.*, 2015). Moreover, these methods cannot account for fine-grained differences in environmental ranges; for instance, a species occurring only within vernal pools is bound to have the same geographic co-ordinates and associated climatic values as one right on the edge of the pool. In the same way, the habitat of cliff-dwelling species cannot be distinguished from that of plants occurring right below or right above the cliffs.

To overcome these limitations, we used a complementary approach consisting of the characterization of environmental ranges of species using traditional floristic habitat assignments. This type of categorization derives from a large set of heuristics that incorporate both abiotic (e.g. climate, soil and fire regime) and biotic (e.g. herbivore pressure and community structure) constraints and has been used by expert field botanists to identify and classify habitat types for >150 years. Therefore, this approach has the advantages of incorporating extensive environmental information within a simple and falsifiable coding, being easily accessible through floristic accounts (Daubenmire, 1966; Haufler *et al.*, 1996). Based on this floristic labelling, we classified species in seven categories (habitats) assumed to have common abiotic (i.e. temperature and water availability) and biotic (fauna) constraints. These habitats were coded with a discrete value as follows: 0 = arctic or alpine tundra; 1 = deserts and semi-deserts; 2 = cliffs and rock faces; 3 = shrubland; 4 = forests; 5 = grasslands; 6 = aquatic. Some taxa could not be unambiguously assigned to a single habitat. In those cases, the relative frequency of occurrence in each habitat type was roughly quantified based on personal observations and using floras and taxonomic treatments when possible, and incorporated in the analyses.

### Ancestral state reconstructions

Habitat evolution in Saxifragales was reconstructed using the supermatrix-based tree and the discrete habitat codes described above. Ancestral habitat reconstructions were performed using a continuous Markov model of discrete character evolution (MkN; Pagel, 1994; Lewis, 2001) employing a Bayesian approximation with diversitree (FitzJohn, 2012). Three different Markov chain Monte Carlo (MCMC) chains were started with priors of rate 0·001, 0·01 and 0·1, respectively, and run for 100 000 steps with exponential prior distributions. Chain convergence was verified using the R package coda (Plummer *et al.*, 2006). All chains converged within the first 1000 generations. Nevertheless, to be conservative, we discarded the first 10 000 steps of every chain and concatenated the last 90 000 steps for each run together to form the posterior probability distributions.

Our phylogenetic tree is almost fully resolved. However, diversitree requires dichotomous trees, so we randomly dichotomized the phylogeny, replacing polytomies with branches of length zero with the function ‘multi2di’ in the ape R package (Paradis *et al.*, 2004). This transformation was relatively small, as only 106 of the 1877 branches had to be replaced and they were all terminal. Another limitation of diversitree is that it cannot incorporate polymorphic data at the tips. To investigate the possible consequences of dichotomizing the tree and the simplification of habitat data, we also reconstructed the ancestral habitat of Saxifragales using Bayesian stochastic mapping (Bollback, 2006) as implemented in the R package phytools (Revell, 2012). We ran SIMMAP (StochastIc Mutational Mapping on Phylogenies; Bollback, 2006) analyses on the original tree for 10 000 iterations and considering both simple and polymorphic habitat coding for all extant species.

### Diversification analyses

Although estimating diversification rates from phylogenetic trees containing only extant diversity is problematic (Quental and Marshall, 2010; Rabosky, 2010; Rabosky and Goldberg, 2015), we decided to employ this frequently used approach to compare rates among habitats. The level of sampling in our tree is relatively high; other than the Daphniphyllaceae (only 4 % of taxa included), all families were represented by at least 21 % (Iteaceae) of their members, with the sampling of the largest families covering at least 25 % of extant diversity (Crassulaceae 365 out of approx. 1450 species, Saxifragaceae 243/approx. 800 species, Grossulariaceae 97/approx. 200 species, Hamamelidaceae 50/approx. 100 species). Therefore, there is no reason to believe that our sampling is biased and we assumed that statistical errors should be neutral and the relative habitat comparisons valid, at least qualitatively (Morlon, 2014).

The influence of habitat associations on the diversification and macroevolutionary dynamics of Saxifragales was investigated using two recently developed methods. First, we used the Cladogenetic State change Speciation and Extinction algorithm (ClaSSE; Goldberg and Igić, 2012) implemented in diversitree to estimate rates of habitat change and extinction. ClaSSE is an extension of the commonly used BiSSE algorithm (Binary Speciation and Extinction; Maddison *et al.*, 2007; FitzJohn, 2012) that allows for cladogenetic character changes by assuming that transitions along the phylogeny may occur either at observed nodes or at nodes where the bifurcation is not observed due to subsequent extinction of one daughter lineage. ClaSSE incorporates this by computing additional speciation rates, *λiij* and *λijj*, in which one of the daughter lineages retains the parent state ‘*i*’ and the other acquires a new state ‘*j*’. Additionally, ClaSSE estimates anagenetic changes as shifts within a single lineage (q01, q10) in a similar way as state-independent models of discrete trait evolution such as MkN (Lewis, 2001; Goldberg and Igić, 2012; Smith and Goldberg, 2015). A full ClaSSE analysis of our data would have required the joint approximation of 245 parameters (all possible speciation rates for seven habitat types and cladogenetic shifts between them: *λiii*; *λiij*; *λijj* plus extinction and anagenetic change rates) with only 950 observations, which would have resulted in unreliable estimates. Thus, we restricted the analyses to allow each ancestral lineage to produce only one daughter in a different habitat, i.e. we only allowed for *λiii*; *λiij* to be approximated and fixed *λijj* = 0.

To estimate the overall rates of anagenetic and cladogenetic transitions into each habitat, we also constructed seven different binary data sets in which taxa were considered to be either present or absent from each habitat, and ran ClaSSE analyses on every data set. We also constrained *λijj* = 0 in these new binary state analyses to make the results comparable with the full ClaSSE analyses. The posterior distribution of the parameters was approximated with Bayesian inference running ten replicates of three MCMC chains of 10 000 generations on the dichotomized ML tree with exponentially distributed priors of rate 0·1, 0·01 and 0·001, respectively, and discarded the first 2000 steps as burn-in.

Additionally, we calculated the rates of speciation, extinction and diversification across the whole tree and investigated whether those rates differed across habitat types or lineages using BAMM (Bayesian Analysis of Macroevolutionary Mixtures) and BAMMTools (Rabosky, 2014; Rabosky *et al.*, 2014). The BAMM results were used to test for differences in trait-dependent speciation, extinction and diversification using the Kruskal–Wallis method in BAMMTools with parameters calculated using 1000 iterations. Additionally, lineage-dependent rates of the three parameters were plotted on the tree to identify regions of the phylogeny associated with significant changes in macroevolutionary rates. Because BAMM requires a fully dichotomous tree with branches of length >0, we assigned all descendants of polytomies in the original tree a branch length of 1/100 000 of the shortest, non-zero branch. We conducted four independent runs of 1 000 000 generations and assessed convergence among chains within and across runs using the logLik estimate provided by the software (Rabosky, 2014).

### Diversification across habitat boundaries

The relative rate of range expansion and habitat specialization of lineages that occurs in environmentally close habitats was estimated with the GeoSSE (Geographic State Speciation and Extinction) algorithm as implemented in diversitree (Goldberg *et al.*, 2011). The rate of per-lineage range expansion from habitat *i* to habitat *j* is represented in GeoSSE by the parameter *d_i_*, and can be taken as a proxy of the capacity of a lineage to spread across habitat boundaries. This parameter is balanced by the per-lineage rate of extirpation within the opposite habitat *x_j_*. The difference between these parameters indicates whether lineages associated with a given habitat ‘*i*’ exhibit a greater tendency to spread into the neighbouring habitat ‘*j*’ (*d_i_* – *x_j_* > 0) or to specialize within their ancestral habitat (*d_i_* – *x_j_* < 0; Goldberg *et al.*, 2011).

We used GeoSSE to estimate the relative habitat specialization of lineages in every pair of habitats that were found to have taxa in common, but focused on the five boundaries of particular eco-evolutionary interest: forest/tundra, grassland/forest, shrubland/forest, grassland/shrubland and shrubland/desert. Because GeoSSE models require that only two habitats be present in the tree, the original dichotomized tree was pruned as needed to leave only the data of the habitat pairs that were compared. GeoSSE analyses were run using an MCMC chain of 100 000 generations with a flat prior rate = 0·1 and discarding the first 2000 generations as burn-in. The significance of GeoSSE results was assessed by determining the proportion of samples from the posterior distribution for which a given result holds, i.e. by estimating in how many samples of the posterior the values of a parameter were within certain boundaries, for instance the proportion of posterior samples in which *d_i_* – *x_j_* > 0. All diversification analyses were corrected for incomplete phylogenetic sampling taking into account that only approx. 50 % of the taxa were included (FitzJohn *et al.*, 2009; Rabosky and Goldberg, 2015).

## RESULTS

### Reconstruction of the ancestral Saxifragales habitat and habitat evolution

The two most common habitats for Saxifragales are forest and cliffs, each with approx. 300 species (30 % of the total; Table 1; Supplementary Data Table S1). However, forest was clearly the most phylogenetically diverse, with 14 of the 15 families represented (all but Penthoraceae). Conversely, desert and tundra habitats included only two families each and 10 % of the species (Table 1). Most species could be unambiguously assigned to a single habitat, and only 113 species (approx. 12 %) had to be assigned to two or more habitats simultaneously. Of these, more than half were found to occur in forest (72 species), shrubland, grassland (61 secies each) or cliffs (49 secies; Table 1). The milder environments (shrubland, forest and grasslands) were thus found to have less specialized species pools.

**Table 1.** Habitat data

Habitat	*n*	Families	ASR	*qi*→	*q*→*i*	Tr. from	Tr. to
Tundra (0)	54/54	Grossulariaceae; Saxifragaceae	0·14/0·00/0·02	0·018/0·054 (2)	0·002/0·006 (2)	10·72	11·28
Desert (1)	33/33	Crassulaceae; Gunneraceae	0·00/0·00/0·00	0·002/0·005 (2)	0·001/0·002 (3)	1·34	1·44
Cliffs (2)	306/355	Crassulaceae; Grossulariaceae; Gunneraceae; Saxifragaceae	0·04/0·01/0·00	0·004/0·013 (4)	0·011/0·054 (1)	16·24	20·76
Shrubland (3)	142/146	Crassulaceae; Grossulariaceae; Haloragaceae; Paeoniaceae; Pterostemonaceae; Saxifragaceae	0·01/0·00/0·00	0·003/0·08 (2)	0·005/0·015 (5)	13·15	11·26
Forest (4)	327/370	Altingiaceae; Aphanopetalaceae; Cercidiphyllaceae; Crassulaceae; Daphniphyllaceae; Grossulariaceae; Gunneraceae; Haloragaceae; Hamamelidaceae; Iteaceae; Paeoniaceae; Penthoraceae; Peridiscaceae; Platanaceae; Saxifragaceae; Tetracarpaeaceae; Trochodendraceae; Vitaceae	0·79/0·99/0·98	0·001/0·006 (1)	0·011/0·046 (1)	24·25	23·39
Grassland (5)	20/41	Crassulaceae; Grossulariaceae; Haloragaceae; Saxifragaceae	0·02/0·00/0·00	0·006/0·015 (3)	0·003/0·015 (1)	5·39	3·24
Aquatic (6)	58/60	Crassulaceae; Gunneraceae; Haloragaceae; Saxifragaceae	0·00/0·00/0·00	0·001/0·002 (5)	0·001/0·003 (5)	1·19	1·10
0 and 2	6	Saxifragaceae					
0 and 4	12	Grossulariaceae; Saxifragaceae					
0 and 2and 4	2	Saxifragaceae					
1 and 3	1	Haloragaceae					
2 and 3	23	Crassulaceae; Haloragaceae					
2 and 3 and 4	3	Crassulaceae; Haloragaceae; Saxifragaceae					
2 and 3 and 5	1	Grossulariaceae					
2 and 4	9	Crassulaceae; Grossulariaceae; Hamamelidaceae; Saxifragaceae					
2 and 5	4	Crassulaceae; Saxifragaceae					
3 and 4	23	Grossulariaceae; Haloragaceae; Paeoniaceae; Saxifragaceae					
3 and 4 and 5	5	Haloragaceae; Paeoniaceae					
3 and 5	5	Haloragaceae; Saxifragaceae					
4 and 5	17	Grossulariaceae; Haloragaceae; Paeoniaceae; Saxifragaceae					
4 and 6	2	Penthoraceae					

Each of the seven habitats considered with their corresponding numeric code in parentheses and sets of habitats in which at least one species occurs. See Table S1 for individual taxon assignment and habitat weights.

*n* = number of taxa assigned to habitat *i* using with simple/‘polymorphic’ (i.e. assigning taxa present in two or more habitats simultaneously to all the habitats) habitat coding. Families present in each habitat.

ASR, probability of each habitat to be that of the MRCA of Saxifragales according to the MkN reconstructions performed with diversitree/the SIMMAP stochastic mapping with ‘simple’ habitat coding/SIMMAP results with ‘polymorphic’ habitat coding.

*qi*→ transition rates from habitat *i* towards other habitats per million years calculated with MkN in diversitree, median rates/maximum rate (habitat associated with maximum rate).

*q*→*i* transition rates towards habitat *i*, median rate/maximum rate (habitat of maximum rate).

Tr. from/Tr. to = mean number of transitions from/to habitat *i* estimated with SIMMAP based on the polymorphic habitat coding.

**Fig. 1.**
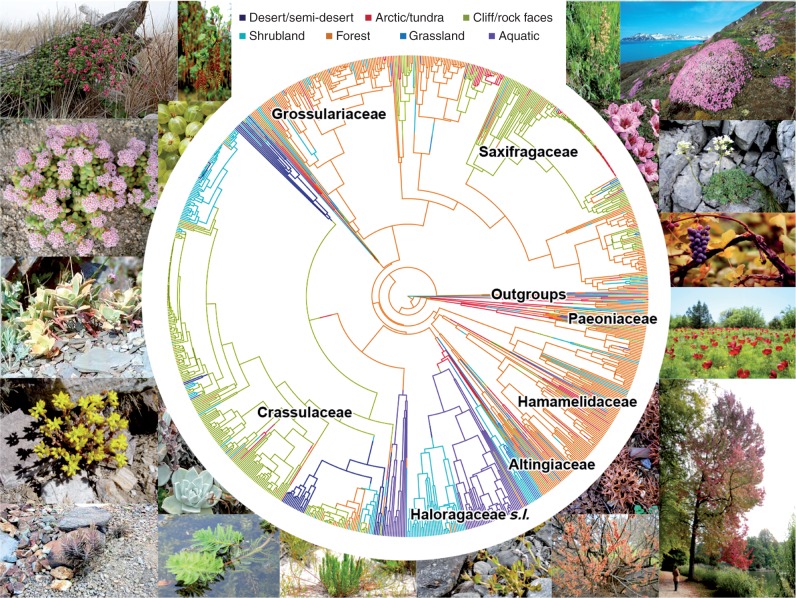
A phylogenetic tree for species of Saxifragales with a reconstruction of habitat evolution using SIMMAP (see key for colours). Representatives of major sub-clades are shown around the tree near the corresponding family name.

Ancestral state reconstructions (ASRs) by the three methods (i.e. MkN and stochastic SIMMAP mapping with and without polymorphic data) yielded similar results, with the differences being more quantitative than qualitative. In every case, the most likely ancestral habitat was forest, with early diversification into aquatic and desert habitats (Fig. 1; Table 1; Supplementary Data Fig. S1). However, transitions from forest into desert or into aquatic habitats were very rare, with median rates of approx. 1 × 10^–4^ and approx. 2 × 10^–4^ per million years, respectively (Supplementary Data Table S2). Although these transitions might have taken place through a succession of habitat states uncorrelated with extant diversity (e.g. Forest → Tundra → Cliff → Desert; Fig. 1), these results underscore that profound evolutionary consequences can derive from exceptional events. In general, MkN models resulted in mean transition rates <0·01, the only exception being the transitions out of tundra and into forest and cliffs habitats (Table 1; Table S2). SIMMAP analyses showed that habitat transitions in and out of forests and cliffs were relatively the most frequent (Table 1; Table S2), indicating that forest and cliff habitats might have been crucial in the evolution of Saxifragales.

### Habitat-dependent diversification

**Fig. 2.**
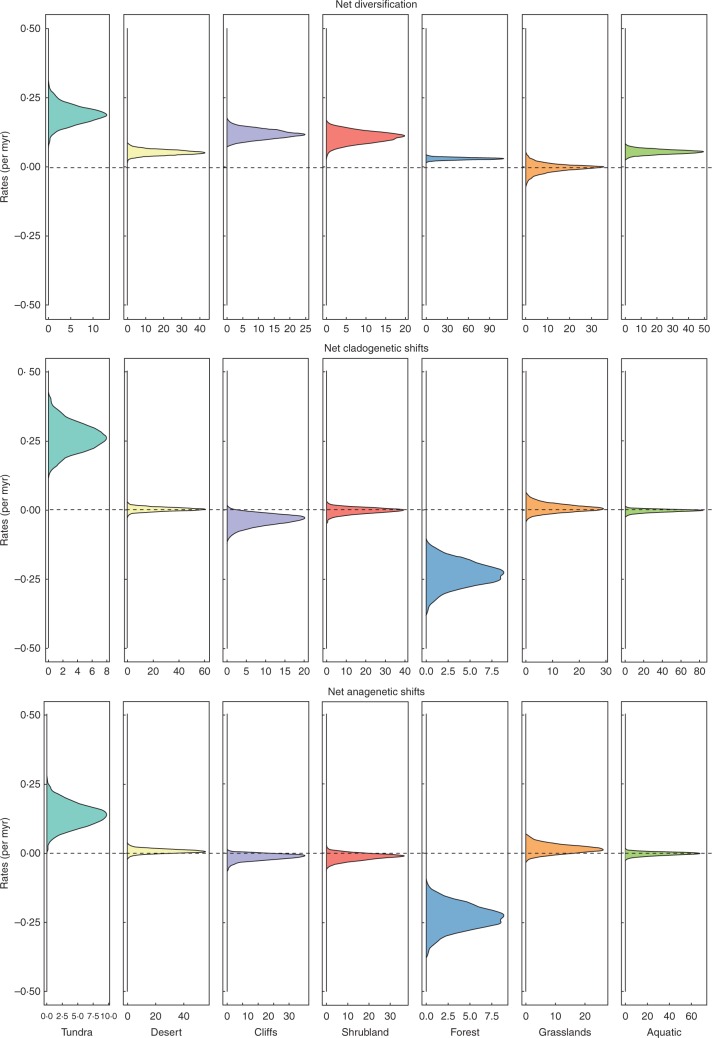
Results of CLaSSE analyses. The plots represent the highest posterior density (HPD) interval values of diversification rates. Intervals overlapping 0 (the dashed line) are not significant. Net diversification: diversification rates without habitat change (i.e. diversification rates when all daughters remain within the ancestral habitat ‘*i*’) computed as the difference between within-habitat speciation and extinction rates; *λiii* – *λiii* – *μi* in the ClaSSE notation. Net cladogenetic shifts: this parameter provides a metric for the relative weight of a habitat as a source or a sink fort extant diversity. It is computed as the difference between cladogenetic speciation out of and into each habitat ‘*i*’. Rates of speciation with a change in habitat (*λiij*) are considered to be the summatory of all cladogenetic speciation events in which lineages from habitat *i* spawn a daughter in a different habitat *j*; i.e. ∑_*i*__=_*_j_^n^ λiij*, and the rates of cladogenetic transition into habitat *j* are computed for each habitat as the *λiij* rate obtained collapsing the data set to a binary situation in which taxa can only be in habitat ‘*i*’ or in an alternative ‘non-*i*’ habitat ‘*j*’. Net anagenetic shifts: the difference of anagenetic transitions within lineages involving habitat shifts from (*qi*→) or towards habitat *i* (*q*→*i*). See text and Table S2 for details.

Taking diversification into account did not seem to alter fundamentally the transition rate results. According to our ClaSSE analyses, habitat transitions, whether anagenetic or cladogenetic, are rare in Saxifragales (Fig. 2; Supplementary Data Table S3). The highest rates were those leading from tundra into forest, whether as the result of a diversification event [cladogenetic habitat change (CHC), Table S3] or as an adaptation (mean anagenetic transition from tundra to forest 0·121 ± 3 × 10^–4^ per million years). These two habitats appeared to have inverse within-habitat diversification patterns: tundra lineages diversified at a very high rate, due to their high speciation rates, whereas diversification in forest environments was very low, largely due to low speciation rates (Fig. 2; Table S3). Tundra seems to be a cradle of recent diversity, while forest habitats appear to drain it (although forests do not seem to constitute a macroevolutionary sink in the strict sense because their diversification rates are positive; Fig. 2). The high diversity found in forests might be at least caused by the recurrent adaptation or expansion of lineages from other habitats, mostly tundra.

Our results also suggested that the cliff/rock faces habitat is highly dynamic from a macroevolutionary standpoint, with the highest rates of speciation, extinction and diversification. Moreover, cliff lineages appeared to transition into forest at a relatively high rate (Table S3). Conversely, diversification and speciation appear to be rare in grasslands, whether within the same habitat or associated with habitat shifts (Fig. 2; Table S3). Grasslands were the only habitat with lower diversification rates than forest, and they also seemed to be relatively easily colonized (Fig. 2; Table S3). However, grassland lineages are species-poor, whereas forest is the most species-rich habitat (Table 1). Desert and aquatic habitats appeared to have comparable diversification patterns, in spite of their clear ecological differences (Figs 1 and 2). These two habitats are relatively species-poor, even though they have been occupied for a long time. These small species pools are probably caused by the low rates of within-habitat diversification and the rarity of lineages from other habitats expanding their range into or adapting to desert or aquatic habitats (Fig. 2; Table S3).

Shrubland is probably the most recently colonized habitat in Saxifragales (Fig. 1; Fig. S1). Perhaps because of this short evolutionary history, the diversification rates of shrubland lineages were high, with frequent speciation and extinction events (Fig. 2; Table S3). The results of BAMM analyses also supported these overall results. Although BAMM did not detect any significant difference in macroevolutionary rates across habitats (*P*-values of 0·158, 0·473 and 0·141 for speciation, extinction and diversification, respectively), plotting of macroevolutionary rates on the tree revealed that the ancient lineages associated with desert (e.g. *Crassula deceptor*–*C. deltoidea* clade, Crassulaceae), aquatic (e.g. *C. mataikona*–*C. peduncularis* clade) and forest habitats (e.g. Altingiaceae, Hamamelidaceae) have the lowest speciation and diversification rates. The highest diversification rates were observed in Crassulaceae and Saxifragaceae (Supplementary Data Fig. S2). In the clade *Graptopetalum paraguayense*–*Echeveria colorata* (Crassulaceae), diversification appears to have taken place within the ancestral cliff habitat. In the other two cases, recent increases in diversification rates were accompanied by the occupation of multiple habitats. In the *Aeonium glandulosum*–*A. ciliatum* clade (Crassulaceae), increased diversification rates resulted in daughters of a likely shrubland species occupying desert and cliffs in addition to the ancestral habitat.

In Saxifragaceae, the group *Saxifraga bicuspidata*–*S. umbellulata* diversified from a cliff-dwelling ancestor and produced taxa occurring in cliffs, grasslands, shrublands and tundra. (Table S1; Figs S1 and S2). However, BAMM results were not completely homogeneous, and intermediate diversification rates could be observed in lineages associated with any habitat (e.g. aquatic *Myriophyllum*, Halogaraceae; desert *Adromischus*, Crassulaceae; forest *Ribes niveum*–*R. divaricatum*, Grossulariaceae; arctic-cliff *Saxifraga tolmiei*–*S. aprica*, Saxifragaceae). Nevertheless, extreme macroevolutionary rates were closely associated with certain habitats: lowest in aquatic, desert and forest habitats and highest in cliffs, shrubland and tundra.

### Macroevolutionary patterns of habitat shift

**Fig. 3.**
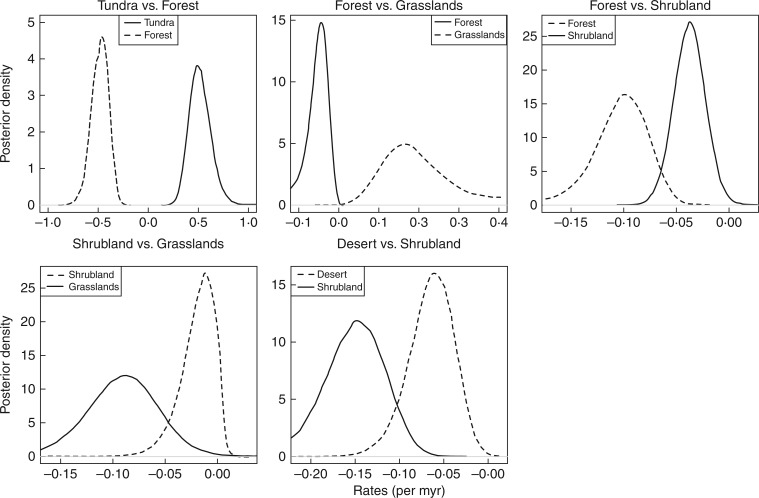
Results of the GeoSSE analyses estimating rates of habitat shift across five focal boundaries. The rate of habitat shift was computed as the difference between the rate of expansion out of the ancestral habitat (*d_i_*) and the rate of extirpation in the alternative habitat (*x_j_*) for each pair of habitats *i*, *j*. Note that expansion of a lineage out of habitat *i* does not necessarily entail abandoning it, but that daughters of that lineage could potentially be present in both habitats, *i* + *j*. Positive values denote expansion of lineages from habitat *i* into habitat *j*, while values < 0 indicate habitat specialization: Forest/Tundra; Grassland/Forest; Shrubland/Forest; Grassland/Shrubland; and Shrubland/Desert. Lines represent the posterior distributions of the *d_i_* – *x_j_* estimated after 100 000 generations with a flat prior rate = 0·1 and discarding the first 2000 generations as burn-in.

The GeoSSE results supported the hypothesis that in Saxifragales most diversification occurs within habitats, and that the rates of environmental range expansion are very low (Fig. 3; Supplementary Data Fig. S3). In the focal ecotones, expansion in environmental tolerance (i.e. *d_i_* – *x_j_* > 0) was only found to be more likely than habitat specialization (i.e. *d_i_* – *x_j_* < 0) in the tundra/forest and grasslands/forest boundaries (Fig. 3). In these two cases, lineages in grasslands or tundra were significantly likely to expand their range into forests (posterior distribution of *d_i_* – *x*_forest_ values not including zero and non-overlapping posterior distributions of *d_i_* – *x*_forest_ and *d*_forest_ – *x_i_*; Fig. 3). In general, forests are always more easily colonized than any other alternative habitats with which they might have taxa in common; there also seems to be a higher likelihood of shrubland lineages to occupy forest than the reverse, although it is not significantly different (*d*_shrubland_ – *x*_forest_ > *d*_forest_ – *x*_shrubland_ with 99·9 % posterior possibility, but posterior distributions are negative and overlap zero; Fig. 3).

Similar results were retrieved in the comparisons of forest–cliff and aquatic–forest habitat boundaries (Fig. S3). Grasslands appeared to be more easily colonized from shrubland than the reverse (*d*_shrubland_ – *x*_grassland_ > *d*_grassland_ – *x*_shrubland_ with 98·4 % posterior probability; Fig. 3). Similarly, the desert–shrubland comparison showed that lineages can transition into desert more easily than out of it (*d*_shrubland_ – *x*_desert_ > *d*_desert_ – *x*_shrubland_ with 99·0 % posterior probability; Fig. 3). However, this case is ambiguous because the range expansion of desert lineages was higher than that of shrubland lineages (mean *d*_desert_ = 0·022 ± 3·4 × 10 ^–5^; mean *d*_shrubland_ = 0·004 ± 10^–5^; and *d*_desert_ > *d*_shrubland_ with posterior probability = 94·1 %), but this was compensated by a significantly higher rate of extirpation in shrubland (mean *x*_desert_ = 0·069 ± 8·4 × 10^–5^; mean *x*_shrubland_ = 0·173 ± 1·1 × 10 ^–4^; and *x*_shrubland_ > *x*_desert_ with posterior probability = 99·5 %). Comparisons across other habitat boundaries further supported the prevalence of habitat specialization. An increase in environmental range expansion was only supported for lineages transitioning from cliff to tundra (Fig. S3). A potential sequence of diversification of Saxifragales lineages might thus be cliff → tundra → forests. In fact, and although cliff and tundra habitats were only reconstructed at relatively recently nodes, several transitions from cliffs into tundra and from either of these habitats into forests were detected in the Saxifragaceae, Grossulariaceae and Crassulaceae (Figs S1 and S2).

## DISCUSSION

### Habitat evolution in Saxifragales and diversification

Our reconstructions indicate that Saxifragales evolved from a forest-inhabiting ancestor. Previous work indicated that the MRCA of this group was probably woody and perennial (Soltis *et al.*, 2013). Peridiscaceae, the sister group to Saxifragales, is a family of trees only found in tropical environments. In view of this, a tropical forest tree or shrub might be posited as a probable ancestor of Saxifragales. Early diversification led to the colonization of desert and aquatic habitats (in the ancestors of Crassulaceae and Halogaraceae), and then the occupation of grasslands (e.g. by ancestral Halogaraceae) and cliffs (e.g. ancestral Saxifragaceae).

We stress, however, that the estimation of trait evolution on phylogenies is prone to error (Mossel, 2003), and caution is needed when interpreting the ASR results. For instance, our analyses of habitat evolution might be mired by the lack of informative fossils. It is always possible that certain lineages might have occurred in a given habitat but that the signal is not retrieved computationally, a bias that can only be corrected based on fossil evidence. Moreover, SIMMAP and MkN analyses did not take into account the incomplete sampling of extant species (approx. 40 % of all Saxifragales), although this is probably less limiting because sampling intensity was largely consistent across the main clades. Notwithstanding these limitations, our results show clearly that the likelihood of a forest lineage spawning a desert or aquatic daughter is extremely small, and yet these transitions appear to lie at the origin of the present diversity of Saxifragales. These findings can be regarded as a clear indication of the profound evolutionary consequences that can derive from relatively unlikely events, such as the colonization of a new habitat.

Soltis *et al.* (2013) found that annual, herbaceous lineages evolved early in Saxifragales. The results presented here indicate that those early transitions in growth form and life history were accompanied by shifts in habitat. Modern desert-inhabiting *Crassula*, *Cotyledon* and *Tylecodon* (Crassulaceae) descend from herbaceous perennial lineages that colonized desert habitats, while lineages that resulted in modern aquatic *Crassula* species appear to have always had an annual life history. This correlated conservatism of habitat types with other traits such as life history might be partly responsible for the generally low transition rates observed among habitat types. Colonizing a new habitat might require significant changes in the biology of a plant, including changes in life form and history (Zanne *et al.*, 2014).

Tundra/alpine and shrubland seem to be the most recently colonized habitats. However, tundra habitats have waned and waxed, to the point of almost complete disappearance throughout Earth’s history. Therefore, tundra lineages might have appeared in the past and became extinct without leaving a detectable trace in the phylogeny, especially since they seem to have a high rate of extinction and to give rise to cliff and especially forest taxa. Alternatively, tundra and shrubland might have been colonized more recently: Mediterranean shrubland has an estimated age of 3·2–2·3 myr (Suc, 1984) while the shrubland-like habitats (i.e. fynbos) in South Africa, which harbour many Saxifragales (e.g. Crassulaceae), are no older than 5 myr (Van Wyck and Smith, 2001). Modern tundra-like environments probably date to the Pliocene and are not older than 7 myr (5·3–2·6 mya; Graham, 2011; Brochmann *et al.*, 2013). Thus, our results might also reflect the relatively recent onset of these habitats and their concomitant colonization by lineages within Saxifragales.

The increased diversification associated with tundra and shrubland is influenced by recent radiations in the Crassulaceae (shrubland) and Saxifragaceae (tundra). In the former, the adaptive radiation of Macaronesian *Aeonium* resulted in a highly diverse clade with elements not only in shrubland but also in desert and cliff habitats and that exhibit a wide variability in growth form, life history and floral morphology (Jorgensen and Olesen, 2001). The other recent radiation of chaparral Crassulaceae in *Graptopetalum* species–*Echeveria* species appears to be associated with the evolution of perennial life forms. Conversely, a rapid increase in diversification in *Saxifraga* was concomitant with the transition into tundra habitats and the emergence of annual, herbaceous life (Soltis *et al.*, 2013). These recent explosive radiations merit further investigation and underscore the importance of tundra and shrubland habitats in Saxifragales.

Additionally, tundra lineages appear to diversify relatively easily into other habitats, namely forest. Rates of within-habitat speciation appeared to be much higher than those resulting in cladogenetic habitat shifts (i.e. habitat colonization of a new environment by a daughter taxon), with the only exception of tundra. Simultaneously, diversification within the forest habitat was particularly low. Nevertheless, this habitat contains the largest number of taxa in our data set. This disparity between diversification and actual diversity might be caused by the frequency with which lineages from other habitats spawn forest taxa, both anagenetically and cladogenetically. Forest diversity appears to be maintained by a frequent contribution from other habitats coupled with low extinction rates.

Besides tundra and shrubland, cliffs provided the habitat with the highest rates of diversification. Additionally, the number of species occurring in cliffs was the highest after forest. Although it is hard to establish a clear causal agent for these patterns, it might be associated with the ephemerality of rock faces, which often collapse and erode, leading to frequent extirpation of populations and therefore increasing the probability of extinction and genetic drift.

Cliffs and forest not only harbour the highest number of species, but also seem to play a core role in diversification. Although the rates of transition out of either of them are low, their high diversity can in time generate daughter lineages that colonize other habitats, compensating for the low probability of any single event. This mechanism might make cliffs and forests ‘evolutionary hubs’ *sensu* Willis *et al.* (2014) that facilitate habitat transitions and determine the overall diversification patterns of the group. Moreover, the two habitats seem to be linked. Cliff lineages appear to expand into forests at a relatively high rate. In the case of tundra, a role for high latitudes as engines of diversification has been proposed for other taxonomic groups such as birds and mammals (Botero *et al.*, 2014), although the reasons for this pattern are unclear. It may be that taxa from highly seasonal environments (i.e. tundra) or where extreme environmental fluctuations are frequent (i.e. cliffs) have a higher colonizing capacity because of their broader environmental tolerances. In any case, the data lead us to posit a scenario for the diversification of Saxifragales consisting of a sequence in which lineages diversify first within cliffs or tundra and then expand into forest, where extinction rates are very low.

### Niche conservatism in Saxifragales

Rates of transition between habitats were generally very low, indicating that habitat shifts either by range expansion or by adaptation to new conditions are rare. Moreover, the tendency of clades to diversify within their ancestral habitats was the norm for Saxifragales. Only cliff, tundra and grassland lineages exhibited a tendency to spread beyond their ancestral habitat, and even in these cases the rates of habitat change were low. This tendency to within-habitat speciation (i.e. cladogenetic specialization) matches the predictions of verbal and mathematical models (Donoghue, 2008; Hua and Wiens, 2013). The association between lineages and their habitats constitutes an evolutionary contingency that leads to a correlation between phylogeny and habitat. This pattern, generally termed niche conservatism, has been posited as a general principle of evolution (Wiens *et al.*, 2010). Our results indicate that the association between certain lineages and habitats is very tight in Saxifragales.

Shifts into tundra, and more particularly desert and aquatic habitats, seemed particularly unlikely. These three habitats were found to have the lowest rates of lineage diversification into them, whether by cladogenetic or anagenetic shift. Conversely, milder habitats such as forests appear to require a lower degree of specialization. According to our data, many taxa occur simultaneously in forest and other environments, and the rates of cladogenetic range expansion and anagenetic shift are highest for lineages expanding into or adapting to forests. Adaptation to the narrow environmental ranges characteristic of extremely arid, cold or aquatic environments entails selection for a wide range of traits, including physiology, life history and anatomy (Meinzer, 2003). Consequently, the gamut of viable phenotypes may be limited and hard to evolve in taxa from other environments.

### Habitat shifts in Saxifragales

Habitat shifts are expected to be relatively rare, but of high evolutionary consequence (Crisp *et al.*, 2009; Donoghue and Edwards, 2014). We focused our analyses on five habitat boundaries that have been influential for the evolution of angiosperms and that are expected to be affected by global climate change: forest/tundra, grassland/forest, shrubland/forest, grassland/shrubland and shrubland/desert. The forest/tundra boundary has been shaped by changes in treeline elevation and the emergence of the current circumpolar flora during the Pliocene. Many extant tundra taxa are believed to be the result of *in situ* diversification or descendants of some Pliocene forest elements (Murray, 1995; Comes and Kadereit, 2003; Brochmann *et al.*, 2013). In Saxifragales, tundra lineages appear to be the result of a relatively high within-habitat diversification rate, and it is more likely for tundra lineages to colonize forest environments than the reverse. Similarly, we hypothesized that the transitions from forests into grasslands or shrubland would be more likely than the reverse, as these shifts had been described as important for the evolution of several plant groups (Verdú *et al.*, 2003; Edwards and Smith, 2010; Bouchenak-Khelladi *et al.*, 2010; Goldberg *et al.*, 2011). However, grassland and shrubland lineages appeared to generate forest taxa more easily than the opposite. Although it seems clear that shifts out of forests have been fundamental for the macroevolution of angiosperms (after all, Saxifragales themselves evolved ‘out of the forest’), our results indicate that expansion into forest from other habitats is also important evolutionarily, at least in qualitative terms.

Historically, changes in precipitation and fire regime have affected the distribution of the grassland/shrubland boundary (Simon *et al.*, 2009; Scheiter *et al.*, 2012). Most grassland lineages appear to be derived from forest or shrubland ancestors, maybe possessing adaptations associated with colonizing open habitats that were crucial for their subsequent diversification (Edwards and Smith, 2010; Lamont *et al.*, 2013). In the case of Saxifragales, it is hard to establish any clear trend, as the shrubland/grassland shift was not statistically supported in either direction. However, our results supported a shrubland to grassland transition as the predominant trend in this clade. Similarly, our data did not support any unambiguous trend in the case of the shrubland/desert boundary, but seemed to indicate that it might have been more likely for shrubland lineages to shift into desert than the opposite. This result is in agreement with patterns described in other systems (Crisp *et al.*, 2009; Heibl and Renner, 2012; Guerrero *et al.*, 2013; although see Lancaster and Kay, 2013). In spite of the limited statistical support, these patterns further underscore the importance of shrublands for the diversification of organisms that can later colonize other habitats.

## Conclusions

The evolution of Saxifragales appears to have been largely coupled with and influenced by habitat characteristics. Most diversification occurs within the ancestral ecological conditions; hence, niche conservatism is of major evolutionarily importance in this clade. Our results indicated that tundra, cliffs and shrubland habitats provide significant sources of diversification, while forests play a central role in the diversification of this group by providing an easily colonized environment with seemingly low extinction rates. The expansion of lineages associated with tundra and grasslands into forests appears to have been particularly significant in Saxifragales. Conversely, transitions into desert and aquatic habitats are very rare, probably because these habitats are highly canalizing. Our results demonstrate that shifts across habitat boundaries are in any case very rare and, consequently, any significant habitat loss or alteration such as those expected under current rates of climate change will have dramatic macroevolutionary consequences for Saxifragales, potentially leading to the extinction of many lineages.

## SUPPLEMENTARY DATA

Supplementary data are available online at www.aob.oxfordjournals.org and consist of the following. Table S1: list of taxa included in analyses with their habitat(s) of occurrence and, in the case of taxa that can occur in more than one habitat, the probability assigned to each habitat. Table S2: transition rates between habitat pairs. The three tables represent the transition rates from habitat ‘*i*’ into habitat ‘*j*’ (*q_ij_*) estimated by SIMMAP stochastic mapping with ‘simple’ habitat coding, SIMMAP with ‘polymorphic’ habitat coding and MkN reconstructions performed with diversitree, respectively. Table S3: detailed results of CLaSSE analyses. Figure S1: results of the ancestral state reconstructions. Figure S2: macroevolutionary rates in Saxifragales. Figure S3: results of the GeoSSE analyses estimating rates of habitat shift across habitat boundaries.

Supplementary Data
