# Supplementary material for: The influence of habitat on the evolution of plants: a case study across Saxifragales
Source: Ann Bot. 2016 Aug 22;118(7):1317–28. doi: 10.1093/aob/mcw160 (PMC5155595; doi:10.1093/aob/mcw160)
Supplement: Supplementary Data [file supp_118_7_1317__index.html]

The influence of habitat on the evolution of plants: a case study across Saxifragales — Supplementary Data 

# The influence of habitat on the evolution of plants: a case study across Saxifragales

## Supplementary Data

files

- Supplementary Data - docx file
- Supplementary Data - csv file
- Supplementary Data - xls file
- Supplementary Data - docx file
- Supplementary Data - tif file
- Supplementary Data - tif file
- Supplementary Data - tif file
