# Supplementary material for: The influence of habitat on the evolution of plants: a case study across Saxifragales
Source: Ann Bot. 2016 Aug 22;118(7):1317–28. doi: 10.1093/aob/mcw160 (PMC5155595; doi:10.1093/aob/mcw160)
Supplement: Supplementary Data [file supp_mcw160_aob-16211-s01.docx]

**Supporting Information**

**Table S1**

List of taxa included in analyses with their habitat(s) of occurrence and, in the case of taxa that can occur in more than one habitat, the probability assigned to each habitat. 0=arctic/tundra; 1=desert/semi-desert; 2=cliffs/rock faces; 3=shrubland; 4=forest; 5=grassland; 6=aquatic

**Table S2**

Transition rates between habitat pairs. The three tables represent the transition rates from habitat “i” into habitat “j” (q_ij_) estimated by SIMMAP stochastic mapping with "simple" habitat coding; SIMMAP with "polymorphic" habitat coding and MkN reconstructions performed with diversitree respectively

**Table S3**

Detailed results of CLaSSE analyses. Mean and highest posterior density (HPD) interval values of the different parameters. Within – habitat speciation: cladogenetic speciation without habitat change values; i.e., speciation rates when all daughters remain within the ancestral habitat i (λiii in the ClaSSE notation). Cladogenetic Habitat Change (CHC): rates of speciation with a change in habitat (λiij) computed as the summatory of all cladogenetic speciation events in which lineages from habitat i spawn a daughter in a different habitat j; i.e., ∑_i=j_^n^ λiij. Speciates into: habitat associated with the maximum CHC. CHC to: rate of cladogenetic transition into habitat j computed for each habitat as the λiij rate obtained collapsing the dataset to a binary situation in which taxa can only be in habitat "j" or in an alternative “non-j” habitat "i" (see text for details). Extinction: extinction rates associated to each habitat type (μi). Within habitat div. : rate of diversification not involving habitat transitions, computed as λiii - μi. Total div.: total diversification rate, including within habitat and cladogenetic diversification rates, i.e., (λiii + CHC) - μi. Anagenetic transitions (AT) from/to: within lineage habitat shifts from (qi→)/ towards habitat i (q→i).

**Fig. S1**

Results of the ancestral state reconstructions. a) MkN results; b) Stochastic mapping (SIMMAP) of simple habitat assignments; c) SIMMAP using polymorphic habitat states.

**Fig. S2**

Macroevolutionary rates in Saxifragales. Each unique colour section of a branch represents the mean of the marginal posterior density of diversification rates at each point in time (cool colours = slow, warm = fast). The colour method used was “Jenks”. Circles denote regions of the phylogeny with maximum marginal rate shift probability (i.e., indicate the branches where accounting for rate shifts leads to a better explanation of the data). The diameter of each circle is proportional to the number of samples in the posterior that is accounted for (i.e., its posterior probability). Arrows point to the exact branches where the shifts have a higher posterior probability. Numbers indicate the branches with significant increases in diversification rates discussed in the text: 1- *Aeonium* spp. (Crassulaceae); 2- *Saxifraga* spp. (Saxifragaceae); 3- *Graptopetalum paraguayense* – *Echeveria colorata* (Crassulaceae)

**Figure S3**

Results of the GeoSSE analyses estimating rates of habitat shift across habitat boundaries. The rate of habitat shift was computed as the difference between the rate of expansion out of the ancestral habitat (d_i_) and the rate of extirpation in the alternative habitat (x_j_) for each pair of habitats i, j. Lines represent the posterior distributions of the d_i_-x_j_ estimated after 100,000 generations with a flat prior rate = 0.1 and discarding the first 2000 generations as burn-in. See main text and Fig. 3 for details.
