# Supplementary material for: The influence of habitat on the evolution of plants: a case study across Saxifragales
Source: Ann Bot. 2016 Aug 22;118(7):1317–28. doi: 10.1093/aob/mcw160 (PMC5155595; doi:10.1093/aob/mcw160)
Supplement: Supplementary Data [file supp_mcw160_aob-16211-s04.docx]

**Table S3**

|  | Tundra | Desert | Cliff | Shrubland | Forest | Grassland | Aquatic |
| --- | --- | --- | --- | --- | --- | --- | --- |
| Within – habitat speciation | 0.202 | 0.059 | 0.220 | 0.143 | 0.032 | 0.006 | 0.064 |
|  | 0.148  0.270 | 0.042  0.078 | 0.184  0.262 | 0.111  0.173 | 0.024  0.040 | 0.000  0.029 | 0.048  0.083 |
| Total Cladogenetic Habitat Change (CHC) | 0.273 | 0.012 | 0.020 | 0.018 | 0.003 | 0.026 | 0.006 |
|  | 0.182  0.374 | 0.004  0.022 | 0.011  0.030 | 0.007  0.029 | 0.001  0.006 | 0.009  0.054 | 0.002  0.012 |
| Maximum CHC | 0.211 | 0.005 | 0.009 | 0.008 | 0.002 | 0.006 | 0.001 |
|  | 0.128  0.298 | 0.001  0.011 | 0.005  0.015 | 0.002  0.014 | 2 x 10^-6^  0.004 | 0.002  0.025 | 0.001  0.005 |
| Speciates into: | Forest | Chaparral | Forest | Cliff | Tundra | Forest | Grassland/Forest |
| CHC to: | 5 x 10^-4^ | 1 x 10^-4^ | 9 x 10^-4^ | 0.002 | 0.007 | 0.002 | 1 x 10^-4^ |
|  | 8 x 10^-8^  0.001 | 3 x 10^-7^  0.002 | 2 x 10^-4^  0.002 | 5 x 10^-4^  0.003 | 0.004  0.009 | 5 x 10^-6^  0.003 | 2 x 10^-8^  2 x 10^-4^ |
| Extinction | 0.010 | 0.005 | 0.102 | 0.034 | 0.002 | 0.010 | 0.008 |
|  | 2 x 10^-6^  0.045 | 1 x 10^-7^  0.022 | 0.044  0.163 | 2 x 10^-6^  0.085 | 3 x 10^-6^  0.008 | 6 x 10^-6^  0.041 | 5 x 10^-7^  0.030 |
| Within habitat div. | 0.188 | 0.051 | 0.117 | 0.107 | 0.029 | -0.003 | 0.054 |
|  | 0.130  0.263 | 0.034  0.070 | 0.086  0.148 | 0.068  0.144 | 0.021  0.037 | -0.042  0.029 | 0.039  0.071 |
| Total div. | 0.461 | 0.064 | 0.137 | 0.125 | 0.033 | 0.024 | 0.060 |
|  | 0.376  0.549 | 0.046  0.085 | 0.104  0.173 | 0.082  0.168 | 0.025  0.040 | -0.017  0.065 | 0.043  0.077 |
| Anagenetic transitions (AT) from: | 0.148 | 0.017 | 0.009 | 0.013 | 0.004 | 0.035 | 0.008 |
|  | 0.075  0.236 | 0.007  0.031 | 0.003  0.016 | 0.005  0.026 | 0.001  0.007 | 0.012  0.068 | 0.003  0.015 |
| AT to: | 8 x 10^-4^ | 8 x 10^-5^ | 3 x 10^-4^ | 6 x 10^-4^ | 5 x 10^-4^ | 0.001 | 1 x 10^-4^ |
|  | 3 x 10  0.002 | 8 x 10^-9^  2 x 10 ^-4^ | 1.5 x 10^-7^  8 x 10 ^-4^ | 4 x 10^-7^  0.001 | 6 x 10^-8^  0.001 | 5 x 10^-8^  0.004 | 2 x 10^-8^  3 x 10^-4^ |
