# Supplementary figures and images for: The influence of habitat on the evolution of plants: a case study across Saxifragales
Source: Ann Bot. 2016 Aug 22;118(7):1317–28. doi: 10.1093/aob/mcw160 (PMC5155595; doi:10.1093/aob/mcw160)

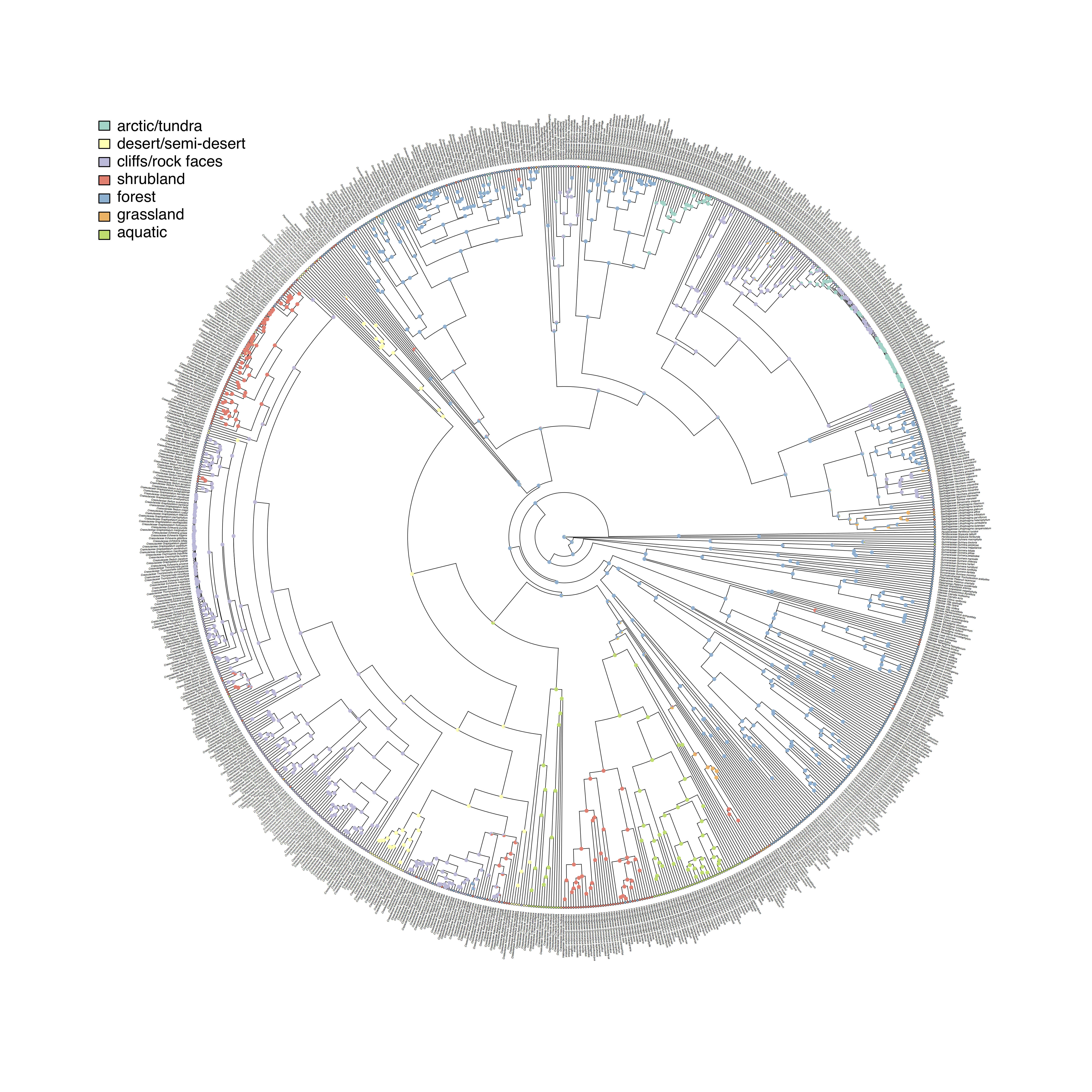

Supplement: Supplementary Data [file supp_mcw160_aob-16211-s05.tif]

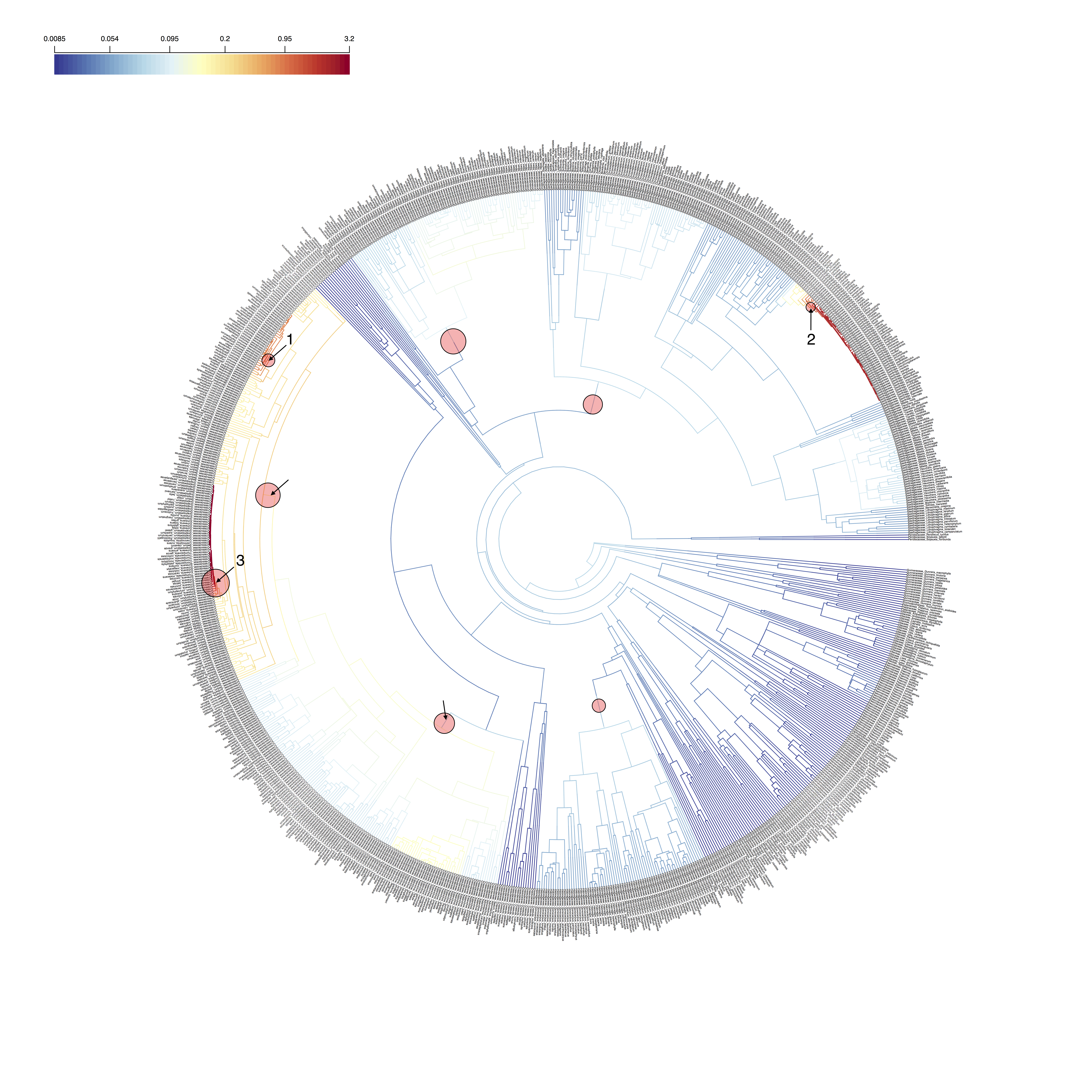

Supplement: Supplementary Data [file supp_mcw160_aob-16211-s06.tif]

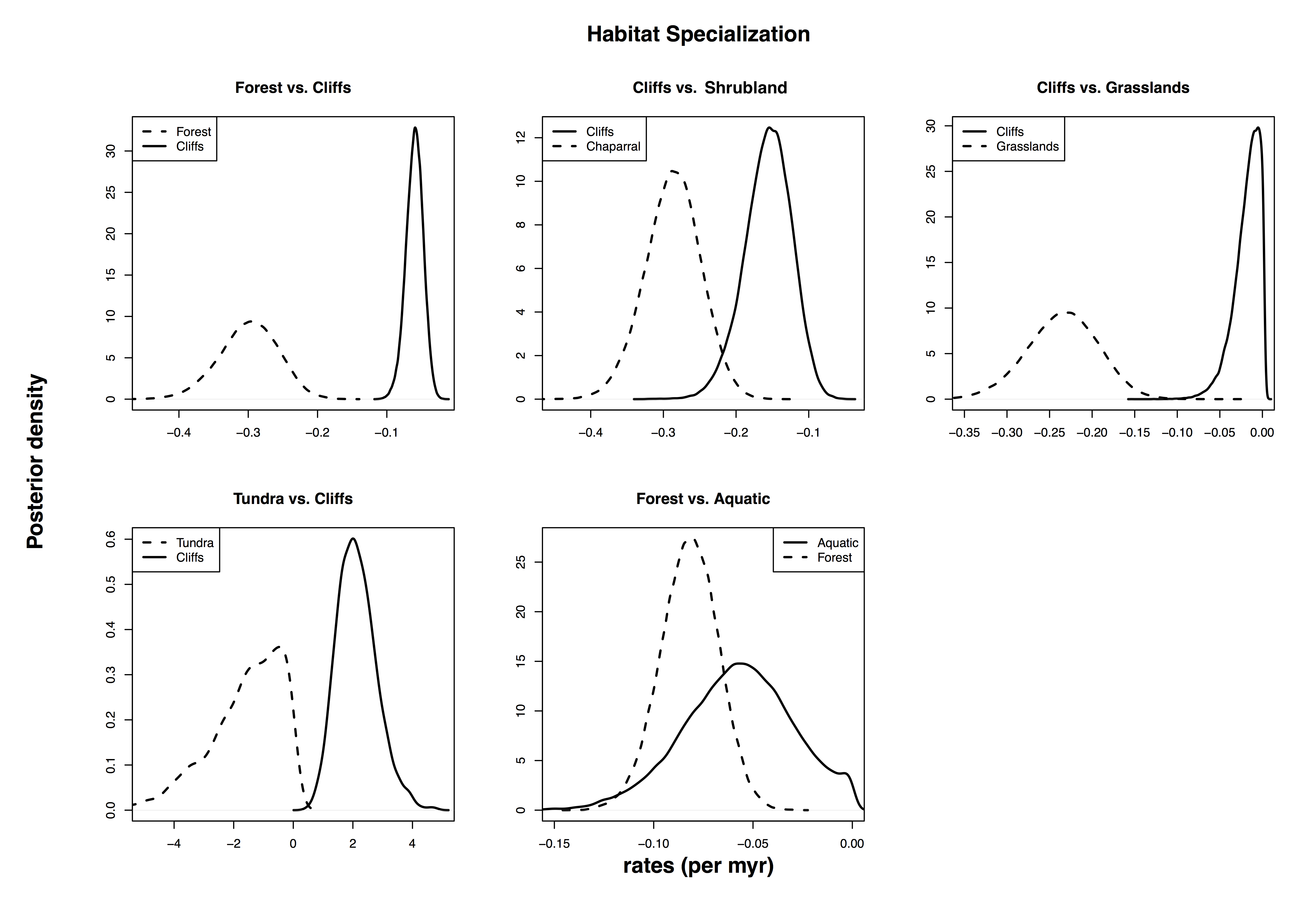

Supplement: Supplementary Data [file supp_mcw160_aob-16211-s07.tif]
